# Recovery of Polyphenols Using Pressurized Hot Water Extraction (PHWE) from Black Rosehip Followed by Encapsulation for Increased Bioaccessibility and Antioxidant Activity

**DOI:** 10.3390/molecules27206807

**Published:** 2022-10-11

**Authors:** Kadriye Nur Kasapoğlu, Evren Demircan, Mine Gültekin-Özgüven, Johanita Kruger, Jan Frank, Ayla Arslaner, Beraat Özçelik

**Affiliations:** 1 Department of Food Engineering, Faculty of Chemical and Metallurgical Engineering, Istanbul Technical University, 34469 Istanbul, Turkey; 2 Institute of Nutritional Sciences, University of Hohenheim, Garbenstraße 28, 70599 Stuttgart, Germany; 3 Department of Food Engineering, Faculty of Engineering, Bayburt University, 69000 Bayburt, Turkey

**Keywords:** green extraction, response surface modeling, *Rosa pimpinellifolia*, in vitro digestion, liposomes, spray drying, chitosan, whey protein

## Abstract

In this work, pressurized hot water extraction (PHWE) of hydrophilic polyphenols from black rosehip fruit was maximized using response surface methodology for simultaneous optimization in terms of extraction yield, total antioxidant capacity, total (poly)phenols, catechin, total monomeric anthocyanins, and cyanidin-3-*O*-glucoside. Extraction parameters, including temperature (X1: 40–80 °C) and the solvent-to-solid ratio (X2: 10–40 mL/g), were investigated as independent variables. Experimentally obtained values were fitted to a second-order polynomial model, and optimal conditions were determined using multiple regression analysis and analysis of variance. The black rosehip extract (BRE) obtained at optimized PHWE conditions was further encapsulated in biopolymer-coated liposomes and spray dried to enhance its processing and digestive stability. After reconstitution, the fabricated particles had an average size of 247–380 nm and a zeta-potential of 15–45 mV. Moreover, encapsulation provided remarkable protection of the phenolics under in vitro gastrointestinal digestion conditions, resulting in up to a 5.6-fold more phenolics in the bioaccessible fraction, which also had 2.9–8.6-fold higher antioxidant activity compared to the nonencapsulated BRE. In conclusion, PHWE in combination with a biopolymer coating is a potent method for the production of stable and safe edible natural extracts for the delivery of (poly)phenolic compounds in food and dietary supplements.

## 1. Introduction

Due to the increased prevalence of degenerative diseases and changes in lifestyle, there has been high consumer demand for functional foods with added health benefits [1,2]. Foods derived from plant origins are cost-effective sources high in numerous bioactive compounds that can be used as value-added ingredients for enrichment purposes. However, to exploit the use of underutilized wild edible plant resources, suitable and convenient extraction methods need to be developed to separate and concentrate the bioactive compounds [3]. Pressurized hot water extraction (PHWE) is an alternative green method which can overcome the disadvantages of conventional organic solvent extraction. Although it is not the most selective extraction technique, PHWE has been receiving more attention for industrial applications due to the benefits of it using water as a solvent, which is nontoxic, recyclable, and readily available, resulting in the process being energy- and time-efficient and low cost [4]. Response surface methodology (RSM) is considered a useful tool in optimizing experimental conditions and simultaneously enabling the maximization of various responses [5].

The *Rosa* genus in the Rosaceae family is widely distributed across Europe, temperate Asia, and North America and includes more than 100 species. Although they have been cultivated since ancient times, some of them can still be found growing in the wild [6]. A variety of bioactive compounds, including vitamin C, vitamin E, and (poly)phenols (see [7] for the used terminology), such as phenolic acids, flavonoids, anthocyanins, proanthocyanidins, and carotenoids have been detected in the *Rosa* species [8]. Unlike other *Rosa* species, black rosehip, the pseudofruits of the *Rosa pimpinellifolia* L. syn. *Rosa spinosissima* L., is known for its dark purplish-black color due to its rich anthocyanin content [9]. Koczka, Stefanovits-Bányai, and Ombódi (2018) reported that the rosehips of *R. spinosissima* had the highest total phenolic content and antioxidant capacity compared to *R. canina*, *R. gallica*, and *R. rugose* [10].

On the other hand, (poly)phenols in food products are highly sensitive to the influence of oxygen, light radiation, enzymes, pH, and heat and, hence, are prone to chemical reactions during food processing and storage, which, in turn, may affect their stability and bioavailability [11]. It is therefore important to preserve their stability by means of encapsulation [12], which furthermore can mask the unwanted bitter and astringent taste of (poly)phenols [13].

To convert black rosehip into a value-added product, several processes have recently been proposed, such as the production of aromatic vinegar [14], tea [15], and yogurt [16] with antioxidant activity or ice cream with improved color stability during storage [17]. However, to the best of our knowledge, no studies have been carried out for the extraction of black rosehip polyphenols by PHWE and liposomal encapsulation. The aim of this work was to optimize the PHWE conditions for the recovery of hydrophilic phenolics and anthocyanins from black rosehip and to convert them into a dry encapsulated form in order to obtain an ingredient suitable for use in functional foods. The impact of encapsulation on the gastrointestinal stability of these (poly)phenols from black rosehip extract and the antioxidant activity of the digested bioaccessible fraction were studied in vitro.

## 2. Results and Discussion

### 2.1. Model Fitting of the Extraction Process

A three level and two factor central composite design was applied to make the response surface optimization for the PHWE of the hydrophilic bioactive compounds from black rosehip (Table 1). The extraction temperature (X1) and solvent-to-solid ratio (X2) were chosen as the two main independent variables, and the measured independent variables in 13 experiments are given in Table 2. Other factors were kept constant throughout all experiments: the pressure of 100 bar and the extraction time of 60 min. Dried whole fruit consisting of flesh and seed parts (5% of residual moisture) was used as solid raw material for the extraction. In the static extraction mode, the solvent-to-solid ratio and moisture content of the feed should be taken into account to obtain better extraction yields [18]. Previous optimization studies for the pressurized liquid extraction or pressurized hot water extraction of plant phenolics demonstrated that both the temperature and the solvent-to-solid ratio were important factors affecting yield [19,20,21].

**Figure 1:**
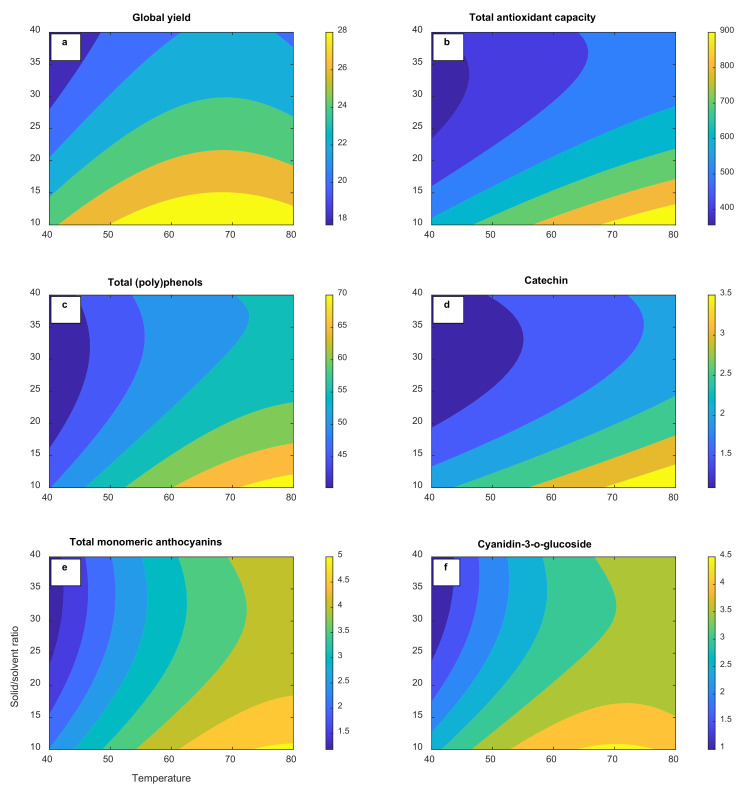
Response surface contour plots for PHW extract of black rosehip showing effects of interactions of temperature and solvent-to-solid ratio on (**a**) global yield (%), (**b**) total antioxidant capacity (mmol TE/g), (**c**) total (poly)phenols (mg GAE/g), (**d**) catechin (mg/g), (**e**) total monomeric anthocyanins (mg cyanidin-3-*O*-glucoside/g), and (**f**) cyanidin-3-*O*-glucoside content (mg/g), in dry basis.

The RSM model was used to generate 3D and contour response surface plots in order to graphically represent the relationship between independent (temperature and solvent-to-solid ratio) and dependent variables (global yield, total antioxidant capacity, total (poly)phenols, catechin, total monomeric anthocyanins, and cyanidin-3-*O*-glucoside). As illustrated in Figure 1a–f, temperature and the solvent-to-solid ratio significantly contribute to all the responses analyzed. The efficiency of the extraction process is quantitatively related to the global yield. Global yields in the range of 17.6–29.6% were achieved under the described operation conditions suggesting that a good amount of the soluble components can be acquired using PHWE (Table 2). Solvent choice affects the extraction efficiency as well as the extract properties. Similar yields (29.7%) have been reported in water extracts in previous studies [22]. The 3D response surface plots can be found in the Supplementary Figure S1.

In the case of the phenolic and anthocyanin compounds, optimization of the temperature is necessary in PHWE in order to take full advantage of the enhanced solubility and mass transfer but also to minimize the thermal degradation [4]. All responses were substantial when the extraction temperature was raised from 40 °C to 80 °C, probably due to the greater extraction efficiency with better mass transfer related to the increase in solubility (*p* < 0.05). Figure 1d–f depicts a slight plateau at temperatures above 75 °C in the response surfaces of the total antioxidant capacity, total monomeric anthocyanins, and cyanidin-3-*O*-glucoside. One explanation for this might be a partial thermal degradation of the anthocyanin major antioxidant compounds in agreement with the previous report of Liazid et al. (2014) [23]. Our preliminary experiments confirmed a 2.2-fold anthocyanin yield decrease when the PHWE process was performed at 100 °C (data not shown). A similar trend has been reported by Koyu et al. (2017) when the temperature augmented from 60 °C to 80 °C during the optimization of the subcritical water extraction of *Morus nigra* L. fruits at 150 bar for 60 min [24]. Hence, an extraction temperature of 75 °C was set as the optimum condition that maximizes the desirability function of the RSM tool for the production of the extract rich in (poly)phenols to be encapsulated as higher temperatures did not confer any rise in yield. As can be seen from Table 3, the model exhibited a significant (*p* < 0.01) relationship between the total (poly)phenols and independent variables, with predicted R^2^ (0.8227) in reasonable agreement with the adjusted R^2^ (0.9454). Considerable interaction effects (*p* < 0.05) among the extraction temperature and solvent-to-solid ratio were also observed on all of the dependent variables analyzed, except global yield, according to the ANOVA analysis.

Another important parameter for the recovery of (poly)phenols is the solvent-to-solid ratio. Minimal consumption of the solvent is the second crucial aspect after maximization of the yield in an industrial scale extraction [25]. The higher solvent-to-solid ratio (*v*/*w*), the lower the amounts of extracted total (poly)phenols and total monomeric anthocyanins were, as can be inferred from the negative significant linear coefficients (*p* < 0.05) in Table 3. The extraction yield is expected to gradually rise with the increasing solid–liquid ratio according to mass transfer principles [26]. However, the results imply that the solute diffusion did not enhance with increasing the solvent-to-solid ratio in our static extraction setting. Previous authors have suggested that a large solvent volume may result in insufficient energy to facilitate cell wall breakage for an efficient extraction of plant secondary metabolites [27]. The extraction yield is also dependent on how the ratio between the solvent and the solid is regulated; that is, keeping the solvent volume or the solid mass constant [25]. In our experimental setting, the solvent-to-solid ratio was adjusted by changing the solid mass while the solvent volume was kept constant. A similar pattern in yield was observed by Clodoveo, Crupi, Muraglia, and Corbo (2022) when the solid–solvent ratio increased in the ultrasound-assisted extraction of polyphenols from carob pods using aqueous ethanol as the solvent [28].

The phenolic and anthocyanin constituents of the black rosehip extract obtained via different conditions of PHWE were in the same range as those previously reported in the literature [15] with slight quantitative differences. Water-soluble total (poly)phenol values in different rosehip species ranged from 150.8 mg to 299.2 mg GAE/100 g in dry weight, of which *R. spinosissima* had the highest (poly)phenolic content as well as antioxidant capacity [10] which is a lower yield than our study. Total monomeric anthocyanins were lower than those found by Odabas and Koca (2021) [9], using microwave-assisted aqueous two-phase extraction with ethanol/ammonium sulfate. The PHW extracts obtained optimized conditions and yielded remarkable total antioxidant capacity values via the CUPRAC assay (Table 4). Pashazadeh, Zannou, Galanakis, Aldawoud, Ibrahim, and Koca (2021) reported DPPH radical scavenging and ferric-reducing antioxidant power results of 110 ± 2 mmol TE/g and 698 ± 15 mmol ISE/g, respectively, for black rosehip using aqueous methanol after the convective drying process. A number of antioxidant capacity assays are present in the literature as they do not correlate well due to differences in the test procedures as well as the surrounding chemistry of the antioxidant compounds [29]. Greater antioxidant potential in our extracts may also be attributed to a possible higher presence of other hydrophilic antioxidant constituents as PHWE is also an efficient method for vitamin C extraction [30]. The spectrophotometric analysis of the PHW extracts revealed total monomeric anthocyanin contents in the range of 1.19–4.98 mg per g of dry fruit (Table 2).

The regression analysis of the experimental data was subjected to a regression coefficient to obtain second-order polynomial equations in terms of coded variables and are given as follows:

(1) $$Global\;Yield=9.536040+0.721555X_{1}-0.486312X_{2}-0.005326X_{1}^{2}+0.004170X_{2}^{2}+0.008775X_{1}X_{2}$$

(2) $$Total\;antioxidant\;capacity=286.755000+18.096500X_{1}-27.274800X_{2}-0.058252X_{1}^{2}+0.533582X_{2}^{2}-0.183282X_{1}X_{2}$$

(3) $$Total\;\left(poly\right)phenols=5.102240+1.781410X_{1}-1.051170X_{2}-0.009473X_{1}^{2}+0.022104X_{2}^{2}-0.0078775X_{1}X_{2}$$

(4) $$Catechin=1.929970+0.046974X_{1}-0.147372X_{2}+0.002750X_{2}^{2}-0.000632X_{1}X_{2}$$

(5) $$Total\;monomeric\;anthocyanins=-3.642710+0.248289X_{1}-0.164783X_{2}-0.001574X_{1}^{2}+0.002062X_{2}^{2}+0.000465X_{1}X_{2}$$

(6) $$\mathrm{Cyanidin-}3\mathrm{-O}\mathrm{-glucoside}=-3.0962710+0.262347X_{1}-0.207847X_{2}-0.001939X_{1}^{2}+0.002168X_{2}^{2}+0.000963X_{1}X_{2}$$

The fitted polynomial equations are presented as contour plots in order to visualize the relationship between the experimental levels of each factor and the response. The results of the ANOVA, the goodness-of-fit, and the adequacy of the models are summarized in Table 3. Considering all response surface models, the “lack of fit” was nonsignificant (*p* > 0.05). The adjusted determination coefficients (R^2^ adj) were also close to R^2^. These findings confirm the adequacy of the model terms to represent the experimental data and to predict the six analyzed parameters. The extraction temperature and solvent-to-solid ratio had significant effects (*p* < 0.05) on the analyzed responses.

Current trends strongly favor the scaling-up of nonconventional green extraction techniques for recovering phenolic compounds that consume less organic solvent(s), involve minimal operational steps, provide high throughput capability, and assure the highest yield at lower costs. Santos and coworkers (2012) reported a 40-fold lower cost of manufacturing, and 2.15- and 1.66-fold more anthocyanins and total phenolic compounds obtained from jabuticaba skins by pressurized liquid extraction using ethanol as a solvent compared to conventional low-pressure solvent extraction [31]. Using pressurized hot water as the solvent of extraction, the PHWE technique is an even greener version of pressurized liquid extraction as water is perhaps the most available naturally occurring liquid on Earth [4,32]. We did not perform an economic evaluation for the feasibility of a large-scale PHWE operation within the scope of this study. However, future studies should focus on the economic feasibility of the global process.

### 2.2. Validation of Optimal Conditions

The main advantage of RSM is the simultaneous maximization of multiple responses in the investigated experimental domain. Based on the above findings, the PHWE conditions giving the best levels of several factors were attained, and they showed that a temperature of 75 °C and a solvent-to-solid ratio of 10:1 mL g^−1^ were optimal, working under 100 bar. The adequacy of the model for predicting optimum yields was experimentally tested using the determined optimized conditions. The optimal extract of BRE revealed mean values of 68.90 ± 3.94, 3.90 ± 0.21, 5.28 ± 0.41, 4.08 ± 0.51 mg, and 930.48 ± 24.37 mmol per g of dry fruit for total (poly)phenols, catechin, total monomeric anthocyanins, cyanidin-3-*O*-glucoside, and total antioxidant capacity, respectively, in the verification experiments. Being similar (*p* > 0.05) to the respective predicted ones, these findings allow validation of the experimental design and demonstrate the effectiveness and good adequacy of the RSM (Table 4). The optimum conditions for extraction were compared to the predicted values of RSM using an independent sample *t*-test. The PHWE process provided a good polyphenol yield with a high antioxidant capacity for a notably shorter extraction time of 60 min, compared to the conventional extraction process. The HPLC analysis showed that cyanidin-3-O-glucoside was the most abundant anthocyanin in all of the PHWE extracts, followed by peonidin-3-O-glucoside, cyanidin-3-O-galactoside, and delphinidin-3-O-glucoside (data not shown) in agreement with the literature data [9].

### 2.3. Characterization of Encapsulated Extract Formulations

Table 5 shows the particle size measurements using the dynamic light scattering technique to characterize the uncoated and coated liposomes in liquid dispersion form before and after spray drying. Nano scale vesicle sizes of the liposomes achieved by the use of microfluidization. The biopolymer coating of the liposomes slightly increased the particle size, and loading BRE into the liposomes resulted in bigger particles (*p* < 0.05). According to the polydispersity index (PDI) as a measure of the width of the particle size distribution, values of between 0.1 and 0.25 prove a narrow size distribution, while values above 0.5 illustrate a very broad size distribution [33]. The relatively high PDI measurements for chitosan-coated samples could be the result of the intrinsic factors of chitosan including PDI, the degree of acetylation, and molecular weight [34].

The addition of maltodextrin prior to spray drying resulted in no alteration in particle size as maltodextrin is an osmotically inactive substance (*p* > 0.05) [35], but it did cause an appreciable increment in the diameter after reconstitution (Table 5). Being a neutral polysaccharide, the effect of the maltodextrin addition on the ζ potential of the liposomal dispersions was also insignificant as expected (*p* > 0.05). By contrast, the ζ potential of the anionic liposomal dispersions (−25.77 ± 1.12 mV) drastically increased to 52.97 ± 2.45 mV and 21.97 ± 0.85 mV by coating oppositely charged chitosan and whey protein, respectively, (*p* < 0.05). On the other hand, the spray-drying process caused a marginal reduction in the ζ potential as is depicted by the values after reconstitution (*p* < 0.05). A high ζ potential and low PDI values are taken as indicators of the physical stability of the nanoparticles [36].

The encapsulation efficiency was satisfactory in all samples in terms of the total (poly)phenols (Table 6). The uncoated BRE liposome, chitosan-coated BRE liposome, and whey protein-coated BRE liposome were found to be 67.2 ± 0.7, 76.7 ± 0.8, and 87.2 ± 4.0, respectively, (*p* < 0.05). Better encapsulation efficiencies were favored in the biopolymer- coated liposomes as reported in other studies [37]. Unlike the freeze-drying process which is disadvantageous in terms of cost and time, spray drying is the most often used process in drug delivery systems for converting aqueous materials into a dried and powder form [38]. However, the retention of the (poly)phenolic compounds during processing is required to preserve the functional properties. High temperatures during spray drying may have a detrimental effect on the thermo-labile (poly)phenols [39]. The total (poly)phenols of the fabricated powders ranged from 55.62 to 61.79 mg per L after reconstitution (Table 6). The amount of (poly)phenols in the samples decreased owing to oxygen and heat exposure during spray drying. The retention efficiency of the total (poly)phenols in the biopolymer-coated liposomal powders was found to be significantly higher than the spray-dried BRE (*p* < 0.05). These results are in agreement with our previous research in which chitosan-coated mulberry extract nanoliposomes spray dried with 20% MD, using the same process conditions, yielded total phenolic and anthocyanin retention efficiencies of 69% and 56%, respectively [40]. In a similar pattern as observed with the retention of phenolics, the retention of the antioxidant capacity of the powders was confirmed with the CUPRAC assay. The chitosan-coated nanoliposomes loaded with flaxseed–peptide fractions retained about 90% and 86% of DPPH and ABTS free radical scavenging activity after spray drying [41].

The physicochemical properties of the fabricated powders are given in Table 7. Moisture content is a critical property of powder products as it indicates the residual water in powders. A range of 1–6% is pursued in the industry for the storage stability of powder [42]. In the current study, the moisture content of all spray-dried samples was below 6% with no significant difference among the samples (*p* > 0.05). Meanwhile, the yield of the powders changed between 60.5% and 72.3%, with a high solubility above 90% (*p* > 0.05) for all of them. Odabaş and Koca (2020) reported similar values for powder yield and solubility that were 52.39 ± 0.65% and 91.14 ± 1.79%, respectively, for spray-dried black rosehip extract microcapsules in maltodextrin–gum arabic (3:1) [43]. 

The color measurements indicated the entrapment of anthocyanins within the biopolymer-coated liposomes, resulting in lower a * values than the spray-dried BRE (*p* < 0.05) in Table 7. The a * coordinate is related to the anthocyanin content in black rosehip fruit that is the pigment responsible for the redness of the fabricated powders. Compared to blank formulations, the chitosan coating also attenuated the whiteness index in the liposomal samples (*p* > 0.05), whereas the whey protein coating yielded a significant decrease (*p* < 0.05). The resultant spray-dried powders had a light pink color, while the blank powders were white, which is also demonstrated by the luminosity (L *) values. The visual appearance of the prepared powders is given in Supplementary Figure S2.

**Figure 2:**
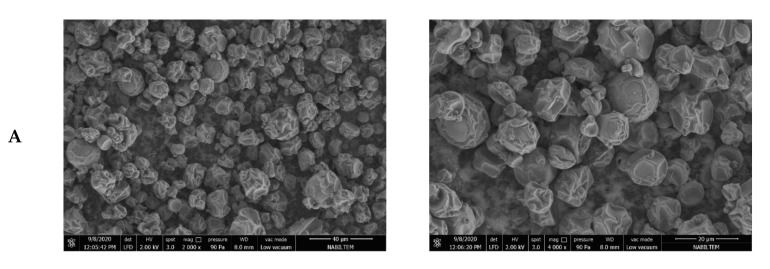

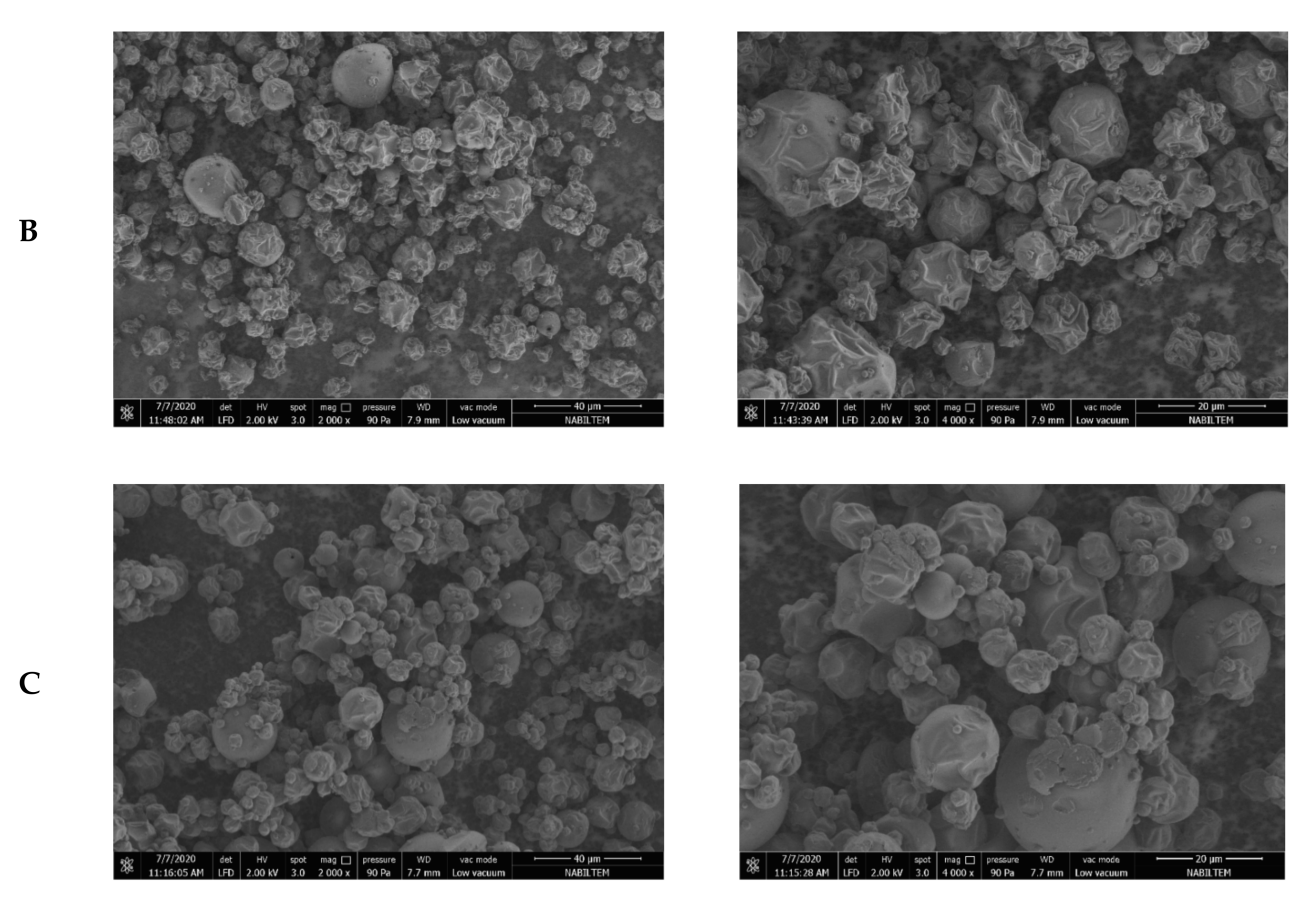
Scanning electron microscopy images of loaded chitosan-coated liposome powder (**A**), loaded whey protein-coated liposome powder (**B**), and water extract powder (**C**). Pictures were taken at 2000× and 4000× magnifications, respectively.

The SEM micrographs of the spray-dried particles of the different formulations are shown in Figure 2. The dried particles exhibited spherical structures with some dents and wrinkles on their surfaces. Either rapid evaporation of water within the particles due to the high inlet temperature or the slow water diffusion rate due to the low inlet temperature cause particle shrinkage during spray drying [44]. The lack of apparent cracks or fissures on the particles in all formulations demonstrate good protection of the internal material encapsulated in the liposomes. The small particles attached to the surface of the capsules might indicate the incomplete encapsulation of the internal material.

### 2.4. Effect of Simulated Digestion on the Total (Poly)phenol and Total Antioxidant Capacity of the Bioaccessibility of Free and Encapsulated Extracts

**Figure 3:**
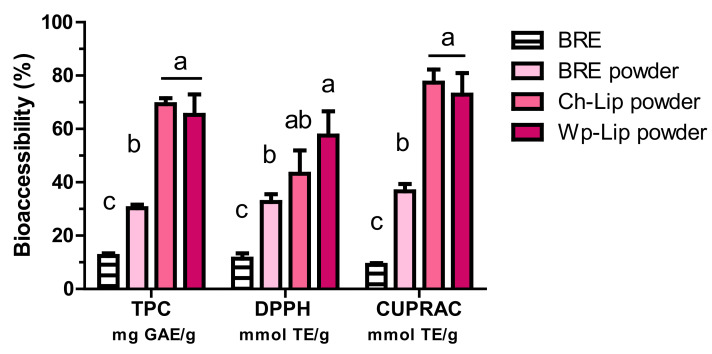
Total (poly)phenolic content (TPC) and total antioxidant capacity (DPPH and CUPRAC assays) (% of TPC and TAC in initial extract) in the bioaccessible fraction of freeze-dried (BRE), spray-dried (BRE powder), and chitosan- and whey protein-coated liposome powdered (Ch-Lip and Wp-Lip) BRE after in vitro gastrointestinal digestion. Values represent means ± SD (*n* = 6). Different superscript letters indicate statistically significant differences (*p* < 0.05).

To determine the total (poly)phenol and total antioxidant capacity in the bioaccessible fraction of the encapsulated black rosehip extract, they were incubated under simulated gastrointestinal conditions. (Poly)phenols are considered bioaccessible when they are released from the food matrix during digestion and solubilized in a form that the enterocytes can absorb [45]. The percentage total of (poly)phenols in the bioaccessible fraction of the chitosan-coated (69.3 ± 2.2%) and whey protein-coated (65.3 ± 9.7%) liposomal extracts were approximately 2.3-fold higher compared to the spray-dried extract (30.3 ± 1.3%) and 5.6-fold higher compared to the nonencapsulated extract (12.4 ± 1.0%) (Figure 3). The total antioxidant capacity in the bioaccessible fraction followed the same trend as the total (poly)phenols for spray-dried BRE, demonstrating an approximate 2.9- and 4.1-fold increase in the radical scavenging activity and cupric ion reducing antioxidant capacity, respectively. Similarly, chitosan- and whey protein-coated liposomal powders had 3.8- and 8.6-fold higher radical scavenging activity and 5.0- and 8.1-fold higher cupric ion reducing antioxidant capacity compared to the nonencapsulated extract after in vitro digestion. These results suggest that the encapsulation process and biopolymer coating greatly enhanced the amount of phenolic and antioxidant activity in the bioaccessible fraction (*p* < 0.05). A similar trend has been previously reported for the liposomal-encapsulated mulberry waste extract by our research group [40]. The application of such delivery systems in dry form yielded better results than in dispersion form [46]. When the liposomal- encapsulated cocoa phenolics in a spray-dried form were incorporated into an Ayran beverage, the bioaccessibility of the cocoa phenolics increased approximately 2.5 fold in comparison with their aqueous dispersion form.

A chitosan coating has been well established to increase the in vitro bioaccessibility of polyphenols in liposomal delivery systems [40,47,48,49]. Recent studies have also reported significant improvements in astaxanthin [50] and curcumin [51] bioaccessibility after the whey protein-coating of liposomes. In our study, while both coatings increased the total (poly)phenol and total antioxidant capacity in the bioaccessible fraction, there was no significant difference between the liposomes coated with chitosan and whey protein (*p* > 0.05). It is noteworthy that this is the first study in which the effect of chitosan and whey protein coating on the bioaccessibility of (poly)phenols was compared. Whey proteins, as a byproduct from cheese or the casein manufacturing process, are well-utilized in the food industry as they are nutritious, widely available, and cheap [52]. In comparison, chitosan, which is obtained through the deacetylation of chitin from crustacean exoskeletons, is a more costly biopolymer (more than one thousand euros per kilogram at Aldrich) despite being a source from abundant marine wastes [53]. However, in our study, the amount of chitosan used for the liposome coating was 10 times less than the needed amount of whey protein indicating no substantial economical difference between both the biopolymers in the coating process.

## 3. Materials and Methods

### 3.1. Materials

Lecithin, 70% phosphatidylcholine from non-GMO soybean (Lipoid P75^®^) was kindly gifted from Lipoid GmbH (Ludwigshafen, Germany). Medium molecular weight chitosan with 80% degree of deacylation was donated by Primex (Siglufjordur, Iceland). Whey protein isolate (WP) with 89% protein content was purchased from Isopure Company, LLC (195 Engineers Road Hauppauge, New York, NY, USA). Maltodextrin with a dextrose equivalent of 16 (Glucidex^®^) was donated (Roquette, France). Triton X-100 was obtained from Merck (Darmstadt, Germany). Sodium acetate trihydrate, acetic acid, sodium hydroxide, hydrochloric acid, potassium chloride, neocuproine, gallic acid, catechin, Trolox, copper (II) chloride, porcine bile extract (B8631), pancreatic lipase (L3126), pancreatin (P3292), and pepsin (P7000) were all from Sigma-Aldrich (Steinheim, Germany). Folin–Ciocalteu’s phenol reagent, ammonium acetate, sodium hydroxide, sodium chloride, sodium nitrite, aluminum chloride, potassium dihydrogen phosphate, and sodium monohydrogen phosphate heptahydrate were purchased from Merck (Darmstadt, Germany). Cyanidin-3-glucoside chloride was obtained from Extrasynthese (Genay, France).

### 3.2. Preparation of Black Rosehip Extract

Black rosehip (*Rosa pimpinellifolia* L.) fruits were obtained from Gümüşhane Province in the Black Sea region of Turkey in 2018. The collected fresh fruits were cleaned to separate the debris and spoiled fruits. After washing, whole fruits were milled using liquid nitrogen and lyophilized for 18 h (Christ Alpha 1-2 LD plus, Buch & Holm, Copenhagen, Denmark). Samples were stored at −20 °C until extraction and analysis. Optimizing the extraction of hydrophilic polyphenolic compounds from black rosehip by pressurized hot water extraction (PHWE) using response surface methodology was conducted in a lab-scale pressurized solvent extraction system (Separex, Champigneulles, France). The sample was placed in 500 mL A40 steel extraction vessel, and deionized and degassed water was pumped using P210 pump maintaining a pressure of 100 bar with no agitation for 60 min. In PHWE, the role of the pressure is to keep water in its liquid phase at the extraction temperature [54]. An extraction period of 60 min was recognized as enough to obtain most of the valuable constituents from plants [55]. The extracts were filtered through Whatman No. 1 paper and stored in glass bottles at 4 °C until analysis.

### 3.3. Experimental Design for RSM Modeling

A central composite design was used to optimize the best extraction conditions to obtain the highest phenolic and anthocyanin content and antioxidant capacity. The central composite design variables under analysis were temperature (X1, 40−60−80 °C) and solvent-to-solid ratio (X2, 10:1−25:1−40:1) (Table 1). The range of values for extraction temperature and solvent-to-solid ratio were selected according to the literature [24,56,57]. The maximum temperature was set to 80 °C as thermal degradation of anthocyanins occurs significantly at higher temperatures [58]. A total of 13 experiments with five replicates at a central point were randomly employed where the investigated responses were extraction yield (Y1), total antioxidant capacity (Y2), total (poly)phenols (Y3), catechin (Y4), total monomeric anthocyanins (Y5), and cyanidin-3-*O*-glucoside (Y6). Dependent variable values were determined in triplicate, and the central composite design data were analyzed using a response surface analysis to fit a second-order polynomial model. Statistical analysis of response surface methodology (RSM) design results was performed with the use of Minitab 16 (Minitab Inc., State College, PA, USA). Three dimensional (3D) and contour response surface graphs were constructed using MATLAB R2020b (MathWorks Inc., Natick, MA, USA).

### 3.4. Global Yield

The collected extracts were put in glass flasks and weighed in analytical balance. Global yield (%, *w*/*w*) was calculated by the mass ratio between extract and feed in dry basis.

### 3.5. Spectrophotometric Assays

#### 3.5.1. Total Phenolic Content

Total (poly)phenols were measured using Folin–Ciocalteu method, as described earlier [46]. Briefly, 0.2 mL of the sample, 1.5 mL of ten-fold diluted Folin–Ciocalteu’s phenol reagent, and then 1.2 mL of sodium carbonate solution (7.5%, *w*/*v*) was mixed at room temperature. After 90 min incubation period protected from light, the absorbance was read at 765 nm in a microplate reader (SynergyHT, Biotek, Winooski, VT, USA). Gallic acid was used for quantification, and the results were expressed in terms of gallic acid equivalent per gram of fruit in dry weight (mg GAE/g dw). All measurements were performed in triplicate for each sample analyzed.

#### 3.5.2. Total Monomeric Anthocyanin Content

Total monomeric anthocyanins were estimated using the pH differential method [59]. Aliquots of each sample were diluted with 0.025 M potassium chloride buffer (pH 1) and 0.4 M sodium acetate (pH 4.5), respectively. The absorbance of each dilution was measured at 520 and 700 nm, respectively. The anthocyanin concentrations were expressed as cyanidin-3-glucoside equivalents (mg/g) = (A × MW × DF × V)/ɛ × W × 0.75, where A = (A520 nm − A700 nm)_pH 1.0_ − (A520 nm − A700 nm)_pH 4.5_, MW = 449.2 g/mol for cyanidin-3-glucoside, DF = dilution factors, V = extract volume, ε =26,900 L/mol extinction coefficient, W = plant sample weight, and 0.75 = pathlength (cm).

#### 3.5.3. Total Antioxidant Capacity (TAC)

Cupric ion reducing antioxidant capacity (CUPRAC) assay was applied [29]. Results were expressed as mmol of Trolox equivalents (TE) per gram of fruit on dry weight (mmol TE/g dw). A 2,2-diphenyl-1-picrylhydrazyl (DPPH) free radical scavenging assay was adapted from previous work [47].

### 3.6. HPLC Analysis of Hydrophilic Compounds

Individual phenolics and anthocyanins in the extracts were detected using a high performance liquid chromatography (HPLC) with a PDA detector (SPD M20A, Schimadzu, Kyoto, Japan). The chromatographic separation of phenolics was performed on an ACE C18 column (250 mm × 4.6 mm, 3 μm) with a guard column (4.0 × 10 mm, 2 μm) (Advanced Chromatography Technologies Ltd., Aberdeen, UK). A gradient of mobile phase A (MQ-water/formic acid, 99.9/0.1 *v*/*v*) and mobile phase B (acetonitrile) was used. The flow rate was 0.5 mL/min, and the injection volume was 10 μL for each standard mixture, and the column temperature was set to 40 °C. A 55 min gradient program was used with the gradient profile as follows: 0–5 min: 10% B, 5–45 min: 55% B, 45–48 min: 90% B, 48–55 min: 10%, and 50–55 min: 10% B.

The chromatographic separation of anthocyanins was performed on a Luna 5μ Phenyl-Hexyl column (250 mm × 4.6 mm, 5 μm) (Phenomenex, Torrance, CA, USA). A gradient of mobile phase A (MQ-water/formic acid, 95:5 *v*/*v*) and mobile phase B (acetonitrile) was used. The flow rate was 0.5 mL/min, and the injection volume was 10 μL for each standard mixture, and the column temperature was set to 40 °C. A 55 min gradient program was used with the gradient profile as follows: 0–40 min: 5% B, 40 min: 30% B, 55 min: 50% B, 57–59 min: 100% B, and 60–65 min: 5%.

### 3.7. Preparation of the Encapsulated Powders

Liposomes were prepared as described earlier by Gültekin-Özgüven et al. (2016) [40]. Lecithin (Lipoid^®^ P75) and lyophilized BRE were mixed in a mass ratio of 10:1 and dissolved in acetate buffer (pH 3.5; 0.1 M) at room temperature. Liposomal dispersions with and without BRE were homogenized by a high shear disperser (DI-25 Yellowline, IKA, Staufen, Germany) at 9500 rpm for 10 min before the aqueous dispersions were passed five times via a high-pressure homogenizer (Microfluidizer Processor M-110L, Microfluidics, Newton, MA, USA) at a homogenization pressure of 25,000 psi. The homogenization chamber was cooled during the homogenization with an ice pack to prevent the heating of samples. Then, negatively charged liposomes were coated by electrostatic deposition of positively charged chitosan (0.8%, *w*/*v*) and whey protein (8.0%, *w*/*v*) layers as optimized in our previous study (data not shown) to avoid depletion flocculation due to less or excessive biopolymer concentrations while coating [37].

Spray-drying encapsulation of the extract was performed by dissolving BRE (0.05%, *w*/*v*) into maltodextrin (20%, *w*/*v*) solution. Similarly, coated liposomal dispersions with and without BRE were also mixed with prehydrated maltodextrin solution in acetate buffer (pH 3.5) while stirring. The resulting liposomal feed consisted of 20% (*w*/*v*) maltodextrin, 0.05% (*w*/*v*) BRE, 0.5% (*w*/*v*) lecithin, and 0.2% (*w*/*v*) chitosan or 2.0% (*w*/*v*) whey protein and dried at a feed rate of 2.5 cm^3^/min and 0.67 m^3^/min air flow using a laboratory scale spray drier equipped with a 1.5-mm nozzle atomizer (mini spray dryer B-290, BUCHI, Flawil, Switzerland). The inlet air temperature was set at 150 ± 5 °C, resulting in an outlet temperature of 90 ± 5 °C. The spray-dried powders were collected, packed in airtight bags, and placed in a desiccator at 4 °C.

### 3.8. Characterization of Prepared Capsule Formulations

#### 3.8.1. Encapsulation and Retention Efficiency

Encapsulation efficiency (EE) of liposomal dispersions was determined using the dialysis technique with few modifications [60]. To remove the nonentrapped BRE phenolics from liposomes, a 5 mL aliquot of liposomal dispersion was placed into 10 kDa dialysis tubing cellulose membrane (Sigma-Aldrich, Seelze, Germany) and immersed in 100 mL of acetate buffer (pH 3.5) with mild stirring, and buffer was refreshed every 2 h during 6 h. Destabilization of liposomes was achieved by the addition of Triton X-100 (0.15%, *w*/*v*) to enable the reagents to reach the entrapped phenolics. The ratio of the content of phenolic compounds before and after dialysis was defined as the encapsulation efficiency in percentage. Unloaded liposomes without BRE were used as blank. To determine the effect of the spray-drying process on total (poly)phenolic content (TPC) and total antioxidant capacity (TAC) of the samples, retention efficiency (RE) was calculated as the ratio of the TPC or TAC in spray-dried powder after reconstitution in acetate buffer (1:10, *w*/*w*) to TPC or TAC in the predried feed solution as a percentage.

#### 3.8.2. Measurement of Particle Size and Zeta (ζ) Potential

The average particle size, polydispersity index, and ζ potential of liposomes was measured by a static light scattering instrument (Mastersizer, 2000; Malvern Instruments, Malvern, UK). Liposome dispersions were diluted 1:10 with acetate buffer solution (pH 3.5) prior to all measurements. The refractive index used for lecithin and aqueous phase was 1.44 and 1.33, respectively. Average particle diameters were reported using the volume mean diameter (d4,3). The spray-dried samples were reconstituted in buffer with a ratio of 1:10 (*w*/*v*). The particle size and ζ potential were also determined after reconstitution as described above.

#### 3.8.3. Morphology

The particle morphology of spray-dried powders was observed using a scanning electron microscope (SEM; ZEISS EVO LS 10, ZEISS, Oberkochen, Germany).

#### 3.8.4. Other Physical Properties

Production yield for the spray-drying process was measured by calculating the mass ratio of the fabricated powder to the total solid content in the feed. Other physical properties of the spray-dried powder, such as moisture content and solubility, were computed using the methods described earlier [61]. The color characteristics (L *, a *, b * values) were measured using a colorimeter (CR 400, Minolta, Osaka, Japan). Whiteness index (WI), hue, and chroma parameters of the samples were calculated accordingly [62].

### 3.9. In Vitro Digestion

The gastrointestinal digestion stability of extract in nonencapsulated and encapsulated forms was assessed (*n* = 6) according to a modified INFOGEST method [63]. For the gastric phase, pepsin was added (1.5 mL 34 mg pepsin/mL 0.1 N HCl–final conc. 5 mg/mL) to 2.5 mg of each phenolic compound (final volume of 8.5 mL), the pH was adjusted to 2, and the digests were incubated in the dark at 37 °C for 1 h (shaking at 120 rpm). For the small intestinal phase, final concentrations of 0.6 mg/mL lipase, 1.2 mg/mL pancreatin, and 3.6 mg/mL bile extract were added (prepared in 100 mM NaHCO_3_) to a final volume of 15 mL. The pH was adjusted to 6.5 using 1 N NaOH, and the digest overlaid with nitrogen gas and incubated, as described above, for 2 h. The final digests were centrifuged (4700× *g*, 60 min, 4 °C) and filtered through 0.2 μm membrane filter (Filtropur S, Sarstedt, Germany) to separate the bioaccessible fraction, respectively. Bioaccessible fraction was overlaid with nitrogen gas and stored at −80 °C before analysis. The total (poly)phenols or total antioxidant capacity was measured in the bioaccessible fraction and is displayed as a percentage of the total (poly)phenol and total antioxidant capacity in the original powders.

### 3.10. Statistical Analysis

Minitab 16 (Minitab Inc., State College, PA, USA) software was used for statistical analysis. All analyses were conducted at least in triplicate. The results were expressed as mean ± standard deviation (SD). The significance (*p* < 0.05) of the differences between the means was determined using one way analysis of variance (ANOVA) with Tukey’s post hoc test.

## 4. Conclusions

In the present study, a combined process for the extraction and the encapsulation of the hydrophilic (poly)phenolic compounds recovered from black rosehip was developed and evaluated. The global polyphenol market was valued at USD 866.41 million in 2020, and it is projected to reach USD 1423.41 million by 2026 according to the report of Mordor Intelligence [64]. To the best of our knowledge, our study was the first for the optimization of the pressurized hot water extraction of black rosehip fruits. A good extraction yield (27.9%) was obtained within 60 min when PHWE was performed with a solvent-to-solid ratio of 10:1 mL/g at 75 °C and 100 bar. The obtained optimized black rosehip extract had a high antioxidant activity (930.5 mmol TE/g dry fruit) due to the presence of the (poly)phenolic compounds (68.9 mg GAE/g dry fruit) and anthocyanins, including cyanidin-3-*O*-glucoside (4.08 mg/g dry fruit). In order to preserve the potentially bioactive compounds during food processing and digestion, BRE-loaded stable dehydrated coated liposomal formulations were successfully fabricated. The in vitro bioaccessibility of the BRE (poly)phenols was augmented approximately 5.6 fold by loading them into coated liposomes with ensured antioxidant activity (*p* < 0.05). Utilizing the most widely used and readily available natural biopolymers, chitosan and whey protein as coatings, liposomes were dried via a spray dryer as a feasible approach for industrial scale production. In conclusion, the developed edible black rosehip extract formulations provide stability to the contained (poly)phenols and preserve their antioxidant activity during digestion and can thus be used to fortify food products with potentially bioactive compounds. Further investigations will be required to explore the effects on individual (poly)phenols in the encapsulated extract during digestion and storage.

## Figures and Tables

**Table 1:** Independent variables and their coded and actual values used for optimization.

Independent Variable	Symbol	Coded Levels		
		−1	0	1
Temperature, °C	X1	40	60	80
Solvent-to-solid ratio, mL/g	X2	10	25	40

**Table 2:** Experimental conditions and results of PHWE on global yield, total antioxidant capacity, total (poly)phenols, catechin, total monomeric anthocyanins, and cyanidin-3-*O*-glucoside in black rosehip fruit, dry basis.

Dependent Variables			Experimental Values					
Run	Temperature (°C)	Solvent–Solid Ratio (mL/g)	Global Yield	Total Antioxidant Capacity	Total (Poly)phenols	Catechin	Total Monomeric Anthocyanins	Cyanidin-3-*O*-Glucoside
1	40 (−1)	40 (1)	17.6	381.41	40.51	1.1	1.19	0.96
2	60 (0)	25 (0)	25.1	514.69	53.45	1.63	3.13	3.26
3	60 (0)	10 (−1)	28.3	892.83	65.61	3.38	4.74	4.1
4	60 (0)	40 (1)	22.3	514.65	54.08	1.85	3.35	3.38
5	60 (0)	25 (0)	24.8	464.29	55.33	2.15	3.77	3.55
6	80 (1)	10 (−1)	29.6	936.98	72.92	4.32	4.98	4.44
7	60 (0)	25 (0)	23.3	513.83	50.91	1.26	3.25	2.97
8	60 (0)	25 (0)	25.2	509.57	52.55	1.73	3.41	3.42
9	80 (1)	25 (0)	24.2	708.84	58.48	2.19	4.25	3.73
10	60 (0)	25 (0)	25.8	606.96	54.31	1.94	3.48	3.18
11	40 (−1)	10 (−1)	26.2	604.02	48.64	2.01	2.3	3.05
12	80 (1)	40 (1)	21.4	514.43	55.46	2.25	4.44	3.51
13	40 (−1)	25 (0)	20.3	411.92	43.68	1.5	1.65	1.22

Results are expressed as mean ± standard deviation (*n* = 3). Global yield (%); total antioxidant capacity (mmol TE/g); total (poly)phenols (mg GAE/g); catechin (mg/g); total monomeric anthocyanins (mg cyanidin-3-*O*-glucoside/g); and cyanidin-3-*O*-glucoside (mg/g). All in dry basis.

**Table 3:** Model summary and analysis of variance (ANOVA) of the effects of extraction conditions on the analyzed responses.

Source	Sum of Squares						Mean of Squares					
	Y1	Y2	Y3	Y4	Y5	Y6	Y1	Y2	Y3	Y4	Y5	Y6
Model	119.91	337.108	817.85	6.80	15.00	11.40	23.98	67.422	163.57	1.70	3.00	2.28
X1 (°C)	20.55	102.155	486.49	2.33	12.13	6.94	20.55	102.155	486.49	2.33	12.13	6.94
X2 (mL/g)	86.71	181.425	229.69	3.09	1.54	2.35	86.71	181.425	229.69	3.09	1.54	2.35
X1^2^	10.16	1.626	11.60	-	0.66	1.12	12.54	1.500	39.66	-	1.09	1.66
X2^2^	2.43	39.809	68.32	1.24	0.59	0.66	2.43	39.809	68.32	1.24	0.59	0.66
X1∙X2	0.06	12.093	21.76	0.14	0.08	0.33	0.06	12.093	21.76	0.14	0.08	0.33
Residual	6.37	23.793	26.89	1.10	0.47	0.51	0.91	3.399	3.84	0.14	0.07	0.07
Lack-of-fit	2.71	12.970	15.46	0.32	0.23	0.31	0.90	4.323	5.15	0.08	0.08	0.10
Pure error	3.65	10.823	11.43	0.78	0.23	0.20	0.91	2.706	2.86	0.19	0.06	0.05
Total	126.27	360.901	844.74	7.90	15.47	11.91						
**Source**	**F Value**						***p*-Value**					
	**Y1**	**Y2**	**Y3**	**Y4**	**Y5**	**Y6**	**Y1**	**Y2**	**Y3**	**Y4**	**Y5**	**Y6**
Model	26.37	19.84	42.58	12.36	44.83	31.48	<0.0001 *	0.001 *	<0.0001 *	0.002 *	<0.0001 *	<0.0001 *
X1 (°C)	22.60	30.05	126.65	16.97	181.19	95.82	0.002 *	0.001 *	<0.0001 *	0.003 *	<0.0001 *	<0.0001 *
X2 (mL/g)	95.34	53.38	59.80	22.45	23.02	32.39	<0.0001 *	0.000 *	<0.0001 *	0.001 *	0.002 *	0.001 *
X1^2^	13.79	0.44	10.32	-	16.35	22.93	0.008 *	0.528	0.015 **	-	0.005 *	0.002 *
X2^2^	2.67	11.71	17.79	8.99	8.88	9.07	0.146	0.011 **	0.004 **	0.017 **	0.021 **	0.020 **
X1∙X2	0.07	3.56	5.67	1.05	1.17	4.61	0.806	0.101	0.049 **	0.336	0.316	0.069
Lack-of-fit	0.99	1.60	1.80	0.41	1.33	2.07	0.482	0.323	0.286	0.793	0.381	0.247
	**Y1**	**Y2**	**Y3**	**Y4**	**Y5**	**Y6**						
R^2^-Sq	0.9496	0.9341	0.9682	0.8607	0.9697	0.9574						
R^2^-Sq (pred)	0.7596	0.6958	0.8227	0.5906	0.8432	0.7126						
R^2^-Sq (adj)	0.9136	0.8870	0.9454	0.7911	0.9481	0.9270						

Independent variables: X1, temperature (°C); X2, solvent-to-solid ratio (mg/mL). Y1, global yield (%); Y2, total antioxidant capacity (mmol TE/g); Y3, total (poly)phenols (mg GAE/g); Y4, catechin (mg/g); Y5, total monomeric anthocyanins (mg cyanidin-3-*O*-glucoside/g); and Y6, cyanidin-3-*O*-glucoside (mg/g) in dry basis. DF: degree of freedom; F-Value: Fisher distribution value; *p*-Value: * significance at *p* < 0.01; ** significance at *p* < 0.05.

**Table 4:** Experimental data for the validation of predicted values at optimal extraction conditions.

	Global Yield	Total Antioxidant Capacity	Total (Poly)phenols	Catechin	Total Monomeric Anthocyanins	Cyanidin-3-*O*-Glucoside
Experimental value	27.90 ± 1.60	930.48 ± 24.37	68.90 ± 3.94	3.90 ± 0.21	5.28 ± 0.41	4.08 ± 0.51
Predicted value	29.5501	946.8044	71.2877	3.9836	5.0344	4.5350
Desirability	0.97750	0.99624	0.94977	0.96245	1.0000	1.0000
*p* *	0.216	0.366	0.403	0.957	0.414	0.669

Abbreviations: global yield (%); total antioxidant capacity (mmol TE/g); total (poly)phenols (mg GAE/g); catechin (mg/g); total monomeric anthocyanins (mg cyanidin-3-*O*-glucoside/g); and cyanidin-3-*O*-glucoside (mg/g). All in dry basis. * Indicate significant differences (*p* < 0.05).

**Table 5:** Average particle diameter, polydispersity index (PDI), and ζ potential of unloaded (blank) liposomes and BRE-loaded liposomes coated with chitosan (0.4 *w*/*v*%) and whey protein (4.0 *w*/*v*%), before and after spray drying (mixed with maltodextrin, 20 *w*/*v*%).

Sample	Particle Size (nm)	PDI	ζ Potential (mV)
Liposome dispersions			
Blank liposome	98.57 ± 2.24 ^gh^	0.205 ± 0.013 ^bc^	−14.50 ± 0.92 ^h^
BRE liposome	114.30 ± 1.61 ^fgh^	0.240 ± 0.007 ^c^	−25.77 ± 1.12 ^i^
Blank Ch-coated liposomal dispersion	158.00 ± 2.27 ^fg^	0.431 ± 0.035 ^abc^	48.97 ± 0.75 ^ab^
Ch-coated liposomal dispersion	194.47 ± 0.60 ^def^	0.480 ± 0.004 ^ab^	52.97 ± 2.45 ^a^
Blank Wp-coated liposomal dispersion	148.07 ± 1.58 ^fg^	0.223 ± 0.004 ^c^	22.17 ± 0.35 ^d^
Wp-coated liposomal dispersion	142.37 ± 2.48 ^fg^	0.241 ± 0.018 ^bc^	21.97 ± 0.85 ^d^
Pre-dried dispersions			
Blank Ch-coated liposome mixed with MD	148.93 ± 3.45 ^fg^	0.479 ± 0.024 ^ab^	50.97 ± 0.75 ^a^
Ch-coated liposome mixed with MD	164.60 ± 2.16 ^efg^	0.553 ± 0.010 ^a^	51.40 ± 1.91 ^a^
Blank Wp-coated liposome mixed with MD	137.83 ± 1.56 ^fgh^	0.237 ± 0.011 ^bc^	18.67 ± 0.81 ^de^
Wp-coated liposome mixed with MD	123.27 ± 0.71 ^fgh^	0.228 ± 0.008 ^c^	21.63 ± 1.17 ^d^
BRE mixed with MD	52.85 ± 20.23 ^h^	0.561 ± 0.309 ^a^	12.40 ± 1.93 ^f^
After reconstitution			
Blank Ch-coated liposomal powder	370.07 ± 45.32 ^ab^	0.569 ± 0.027 ^a^	42.57 ± 2.81 ^c^
Ch-coated liposomal powder	384.33 ± 25.22 ^a^	0.560 ± 0.049 ^a^	45.70 ± 1.70 ^bc^
Blank Wp-coated liposomal powder	254.84 ± 57.12 ^cd^	0.411 ± 0.034 ^abc^	15.43 ± 1.02 ^ef^
Wp-coated liposomal powder	247.33 ± 52.42 ^cde^	0.247 ± 0.013 ^bc^	16.93 ± 0.67 ^e^
BRE powder	284.73 ± 64.12 ^bc^	0.250 ± 0.058 ^bc^	7.05 ± 2.00 ^g^

Results expressed as mean ± SD (*n* = 3). Ch: chitosan; Wp: whey protein; MD: maltodextrin; and BRE: black rosehip extract obtained at optimal PHWE conditions. Different superscript letters in columns indicate statistically significant differences between samples (*p* < 0.05).

**Table 6:** Encapsulation efficiency (EE) and retention efficiency (RE) in terms of total (poly)phenolic content (TPC) and total antioxidant capacity (TAC) of liposomal dispersions prepared with black rosehip extract (BRE).

Formulation	Assay			
	Folin–Ciocalteu		CUPRAC	
	TPC	EE (%)	TAC	EE (%)
Liposomal dispersion	329.71 ± 0.92 ^a^	67.2 ± 0.7 ^c^	827.41 ± 16.53 ^a^	60.4 ± 1.1 ^b^
Ch-coated liposomal dispersion	161.80 ± 1.39 ^b^	76.7 ± 0.8 ^b^	293.50 ± 25.5 ^c^	69.0 ± 4.6 ^ab^
Wp-coated liposomal dispersion	124.98 ± 7.48 ^c^	87.2 ± 4.0 ^a^	346.61 ± 34.17 ^b^	70.4 ± 4.4 ^a^
		**RE (%)**		**RE (%)**
Ch-coated liposomal powder	59.22 ± 2.10 ^d^	62.9 ± 1.5 ^a^	114.15 ± 18.95 ^f^	69.8 ± 4.1 ^a^
Wp-coated liposomal powder	61.79 ± 2.70 ^d^	65.6 ± 2.1 ^a^	124.89 ± 8.93 ^d^	72.2 ± 2.4 ^a^
BRE powder	55.62 ± 1.54 ^d^	56.4 ± 1.8 ^b^	118.62 ± 1.09 ^e^	63.9 ± 4.9 ^a^

Results expressed as mean ± SD (*n* = 3). Ch: chitosan; Wp: whey protein; TPC (mg GAE/L); and TAC (mg TE/L). Different superscript letters in columns indicate statistically significant differences between samples (*p* < 0.05).

**Table 7:** Characterization of spray-dried black rosehip extract (BRE) and BRE-loaded liposomal powders.

Formulation	Moisture Content (%)		Process Yield (%)		Solubility (%)	
Blank Ch-coated liposomal powder	5.0 ± 0.6		72.3 ± 1.2 ^a^		94.3 ± 1.2	
Ch-coated liposomal powder	5.2 ± 0.9		60.5 ± 2.7 ^b^		95.4 ± 0.3	
Blank Wp-coated liposomal powder	4.6 ± 1.0		70.9 ± 1.1 ^a^		93.9 ± 4.7	
Wp-coated liposomal powder	5.9 ± 0.8		62.0 ± 5.1 ^b^		96.3 ± 4.0	
BRE powder	5.2 ± 0.8		61.5 ± 2.4 ^b^		98.6 ± 5.3	
**Formulation**	**WI (%)**	L *	a *	b *	**Chroma**	**Hue Angle**
Blank Ch-coated liposomal powder	95.85 ± 0.76 ^ab^	96.14 ± 1.34 ^c^	2.94 ± 0.11 ^b^	0.28 ± 0.18 ^c^	2.97 ± 0.10 ^b^	5.48 ± 3.59 ^d^
Ch-coated liposomal powder	95.33 ± 0.24 ^b^	98.33 ± 0.78 ^ab^	−0.49 ± 0.08 ^c^	3.76 ± 0.83 ^b^	3.79 ± 0.83 ^b^	97.47 ± 0.38 ^a^
Blank Wp-coated liposomal powder	97.12 ± 0.04 ^a^	96.57 ± 0.16 ^bc^	2.33 ± 0.12 ^d^	2.14 ± 0.16 ^a^	3.17 ± 0.19 ^b^	42.21 ± 1.07 ^b^
Wp-coated liposomal powder	92.10 ± 0.59 ^c^	99.62 ± 0.04 ^a^	−0.30 ± 0.01 ^d^	2.84 ± 0.07 ^b^	2.86 ± 0.06 ^b^	95.87 ± 0.28 ^a^
BRE powder	95.09 ± 1.06 ^b^	95.32 ± 0.52 ^c^	5.94 ± 0.58 ^a^	2.26 ± 0.34 ^b^	6.36 ± 0.66 ^a^	20.79 ± 1.07 ^c^

Results expressed as mean ± SD (*n* = 3). Ch: chitosan; Wp: whey protein; WI: whiteness index; L *: luminosity; a *: red/green value; and b *: blue/yellow value. Different superscript letters in columns indicate statistically significant differences between samples (*p* < 0.05).

## Data Availability

Data are contained within the article or Supplementary Materials.

## Supplementary Materials

The following supporting information can be downloaded at https://www.mdpi.com/article/10.3390/molecules27206807/s1: Figure S1: Three-dimensional response surface plots for PHWE of black rosehip showing effects of interactions of temperature and solvent-to-solid ratio on (A) global yield (%), (B) total antioxidant capacity (mmol TE/g), (C) total (poly)phenols (mg GAE/g), (D) catechin (mg/g), (E) total monomeric anthocyanins (mg cyanidin-3-O-glucoside/g), and (F) cyanidin-3-O-glucoside content (mg/g), in dry basis; Figure S2: Visual appearance of powders; loaded chitosan-coated liposome (A), loaded whey protein-coated liposome (B), extract powder (C), chitosan-coated control liposome (D), and whey protein-coated control liposome (E); Figure S3: Visual appearance of powders after reconstitution in distilled water; extract powder (A), chitosan-coated control liposome (B), loaded chitosan-coated liposome (C), whey protein-coated control liposome (D), and loaded whey protein-coated liposome (E); Table S1: Linear regression data and quality control parameters used in HPLC quantification.
