# Supplementary material for: Recovery of Polyphenols Using Pressurized Hot Water Extraction (PHWE) from Black Rosehip Followed by Encapsulation for Increased Bioaccessibility and Antioxidant Activity
Source: Molecules. 2022 Oct 11;27(20):6807. doi: 10.3390/molecules27206807 (PMC9610414; doi:10.3390/molecules27206807)
Supplement: Supplementary file 1 [file molecules-27-06807-s001.zip › molecules-1939379-supplementary.pdf]

Supplementary Materials

Supplementary Tables:

Table S1. Linear regression data and quality control parameters used in ~~UF~~HPLC quantification.

Compound	HPLC-DAD λmax (nm)	Rt.(min)	Regression equations (x is the content; y is the peak area)	R^2
Catechin	280	22.57	Y = 8846.02X + 4326.11	0.9983
Cyanidin-3-*O*-glucoside	520	33.7	Y = (3.64861e-005)X + (-4.98058)	0.9977

Supplementary Figures:

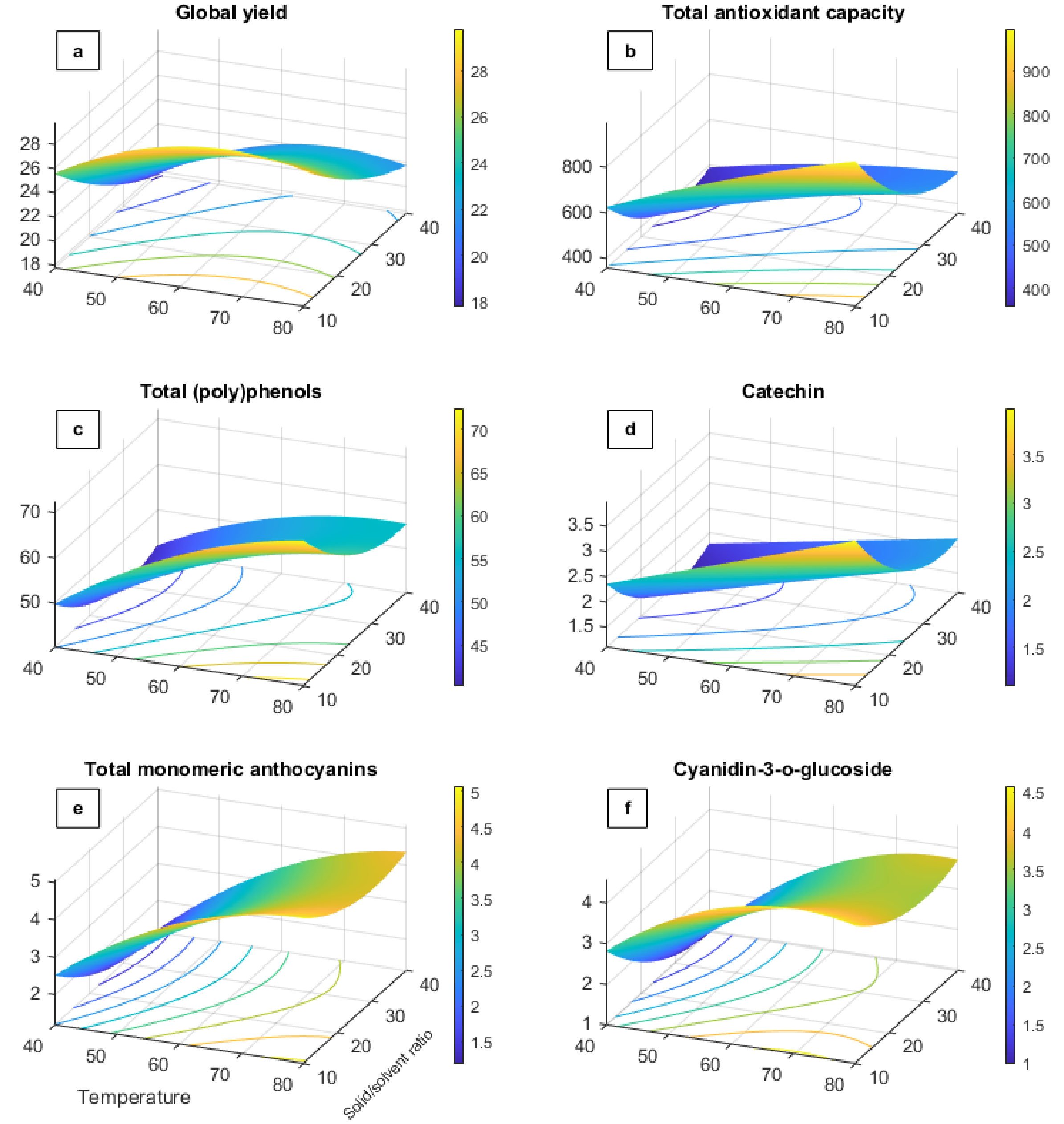

Figure S1. Three-dimensional response surface plots for PHWE of black rosehip showing effects of interactions of temperature and solvent-to-solid ratio on (A) global yield (%), (B) total antioxidant capacity (mmol TE/g), (C) total (poly)phenols (mg GAE/g), (D) catechin (mg/g), (E) total monomeric anthocyanins (mg cyanidin-3-*O*-glucoside/g), and (F) cyanidin-3-*O*-glucoside content (mg/g), in dry basis.

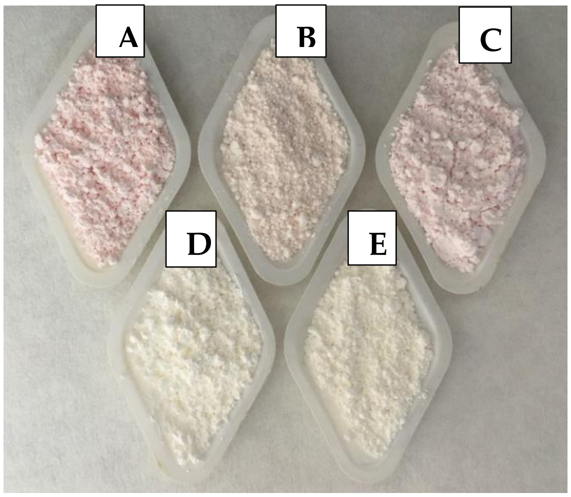

Figure S2. Visual appearance of powders; loaded chitosan-coated liposome (A), loaded whey protein-coated liposome (B), extract powder (C), chitosan-coated control liposome (D), whey protein-coated control liposome (E).

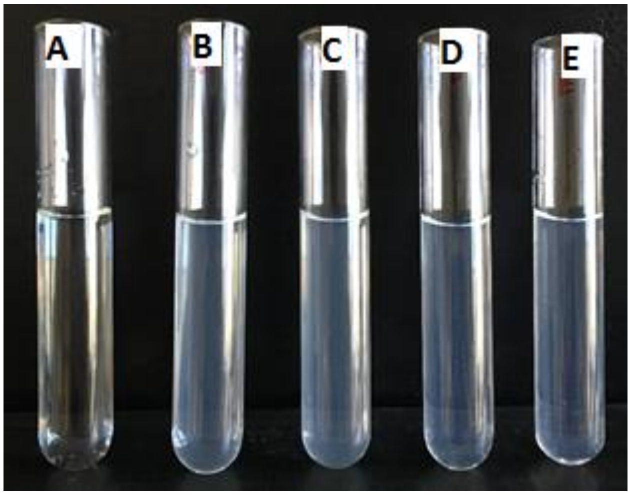

Figure S3. Visual appearance of powders after reconstitution in distilled water; extract powder (A), chitosan-coated control liposome (B), loaded chitosan-coated liposome (C), whey protein-coated control liposome (D), loaded whey protein-coated liposome (E).
